# Supplementary material for: Polymeric Carbon Nitrides for Photoelectrochemical Applications: Ring Opening-Induced Degradation
Source: Nanomaterials (Basel). 2023 Mar 31;13(7):1248. doi: 10.3390/nano13071248 (PMC10097008; doi:10.3390/nano13071248)
Supplement: Supplementary file 1 [file nanomaterials-13-01248-s001.zip › nanomaterials-2278794-supplementary.pdf]

Supporting Information

Ring opening induced photoelectrochemical degradation of polymeric carbon nitrides

Florentina Iuliana Maxim [1], Eugenia Tanasa [2], Bogdan Mitrea [1], Cornelia Diac [1], Tomáš Skála [3], Liviu Cristian Tanase [4], Cătălin Ianăși [5], Adrian Ciocanea [6], Stefan Antohe [7,8], Eugeniu Vasile [2], Eugenia Fagadar-Cosma [5] and Serban N. Stamatin [1,7,*]

[1] 3Nano-SAE Research Centre, University of Bucharest, Atomistilor 405, 077125 Magurele, Romania
[2] Department of Oxide Materials and Nanomaterials, Faculty of Applied Chemistry and Material Science, University POLITEHNICA of Bucharest, 060042 Bucharest, Romania
[3] Department of Surface and Plasma Science, Charles University, V Holešovičkách 2, 18000 Prague, Czech Republic
[4] National Institute of Materials Physics, Atomistilor 405A, 077125 Magurele, Romania
[5] "Coriolan Drăgulescu" Institute of Chemistry, Mihai Viteazul Ave. 24, 300223 Timisoara, Romania
[6] Hydraulics and Environmental Engineering Department, University POLITEHNICA of Bucharest, 060042 Bucharest, Romania
[7] Faculty of Physics, University of Bucharest, Atomistilor 405, 077125 Magurele, Romania
[8] Academy of Romanian Scientists (AOSR), Ilfov No 3, 050094, Bucharest, Romania
* Correspondence: serban.stamatin@unibuc.ro

Materials and Methods

Reagents

$NaHCO_3$ (99%, S.C. Herodio Crafts S.R.L., Romania), KCl (99%, Chimactiv S.R.L., Romania), $K_3[Fe(CN)_6]$ (99%, Synteza, Poland), melamine (99%, Sigma Aldrich, USA) and ethanol (99%, S.C. Tunic Prod S.R.L., Romania) were used without further purification. Glass slides coated with a fluorine doped oxide (FTO) with a surface resistivity of 7 Ω cm^{-2} was supplied by Sigma-Aldrich (#735167-1EA, St. Louis, Missouri, USA), then cut into 3 cm x 3 cm pieces. A Milli-Q DirectQSystem (Burlington, MA, USA) was used for obtaining ultrapure water (> 18.2 MΩ cm).

g-CN and g-CN-HT synthesis

Metal free carbonitride (g-CN) was prepared by thermal polycondensation of melamine at 550°C for 4 hours. The precursor was placed in a covered alumina crucible, in air, and heated to 550°C with a 5°C min^{-1} heating rate. After the synthesis, the crucible was freely cooled to room temperature. Annealed graphitic carbon nitride (g-CN-HT) was obtained by annealing g-CN for 4 hours at 675°C under Ar atmosphere. The final products had a yellow (g-CN) and a red (g-CN-HT) color.

Structural and morphological characterization

Photoemission and X-ray absorption experiments were carried out at the Materials Science Beamline at Elettra Synchrotron facility in Trieste, Italy. It is a bending-magnet beamline with a tuning range 22-1000 eV. An ultra-high vacuum experimental chamber (base pressure below 2×10-10 mbar), a hemispherical electron analyzer (Specs Phoibos 150), sample manipulator and load lock were used to conduct the experiments. Photoemission measurements were performed at normal emission (60° incidence) geometry with photon energies 700 eV (core levels O 1s, N 1s, C 1s; total resolution 0.5-0.7 eV). Changes in the core level spectra were caused by the synchrotron radiation during photoelectron emission experiments. XPS measurements are shown only with the Al Kα (1487 eV, total resolution 1eV). The photocurrent generated by a gold mesh was used to normalize the spectra. X-ray absorption (NEXAFS) measurements were performed in the Auger-electron yield mode near the C and N K-edges. The resolution was 0.3-0.4 eV. The energy scale was calibrated using data measured on an Ar-sputtered gold foil. Reference data from this sample were also used for intensity normalization of the NEXAFS spectra.

Morphological characterization was done on a scanning electron microscope (SEM) and transmission electron microscope (TEM) operated at 30kV and 300 keV, respectively. The SEM (Quanta Inspect F) and TEM (Tecnai G2 F30 S-Twin) were both manufactured by FEI-Philips (Hillsboro, OR, USA). The pore size distribution, specific surface area and total pore volume were evaluated by Quantachrome Nova 1200e (Anton Paar, St Albans, UK). The samples were initially degassed at 200 °C for 15 hours and analyzed with nitrogen at 77K. The BET method was used for determining the total specific surface area. For the pore size distribution, BJH method (Barrett, Joyner, and Halenda) was applied. Using the last point of isotherms from adsorption, the total pore volume was determined. A Panalytical X'PERT PRO (Almelo, The Netherlands) was used to carry out powder X-ray diffraction (Cu Kα = 1.54 Å). UV-Vis

spectroscopy was done by means of a Jasco spectrophotometer (V550, Tokyo, Japan). FT-IR spectra were acquired on a Jasco FT-IR 6200 spectrophotometer on IR grade KBr pellets (Acros Organics, USA).

Electrochemical measurements

Inks, containing 50 mg carbon nitride powder in 5 mL ethanol, were homogenized for 15 min using an ultrasonic processor (Ultrasonics FS-250N, MXBAOHENG, Zhejiang, China). The inks were sprayed on heated (80 °C) FTO glass plates (working electrode support). Polymeric carbon nitride films were obtained by means of an air brush operated at 2 bar. The air brush was manufactured by HSENG, China, model AF186 Mini Air Compressor.

Carbon dioxide reduction reaction and Mott – Schottky measurements were measured by means of a Voltalab electrochemical workstation (Voltalab, PGZ 301, Radiometer, Copenhagen). The photo-electrochemical cell (PECC-2) was manufactured by ZAHNER in Germany. The photo-electrochemical cell was equipped with a transparent working electrode with an 18 mm optical window diameter. A mercury lamp (PS-2, China) was used for front illumination. FTO glass deposited with various g-CN served as a working electrode. A platinum wire was used as a counter electrode. The reference electrode, Ag/AgCl, potential was measured against the standard hydrogen electrode. The reference electrode potential was measured 5 times with an average of 0.201 V +/- 0.001 V (95% confidence interval). Counter and reference electrodes were made in-house.

Photoelectrochemical degradation under visible light was tested on the g-CN and g-CN-HT. CO_2 was purged in the PEC cell filled with 0.1 M $NaHCO_3$ for twenty minutes before the electrochemical measurements. The reduction photocurrents of each sample were measured under different applied potentials (– 0.3 V, – 0.4 V, – 0.5 V, – 0.6 V, – 0.7 V). Each measurement consisted of 4 cycles and each illumination cycle had 30 seconds dark and 30 seconds illuminated.

Mott-Schottky measurements were based on the determination of the inverse squared capacitance by electrochemical impedance spectroscopy in 0.1 M KCl with 1 mM $K_3Fe(CN)_6$. Dynamic electrochemical impedance spectroscopy was performed between –1 V and –2 V range vs. Ag/AgCl at a fixed frequency of 100 kHz, 10 mV amplitude and a potential step of 0.025 V.

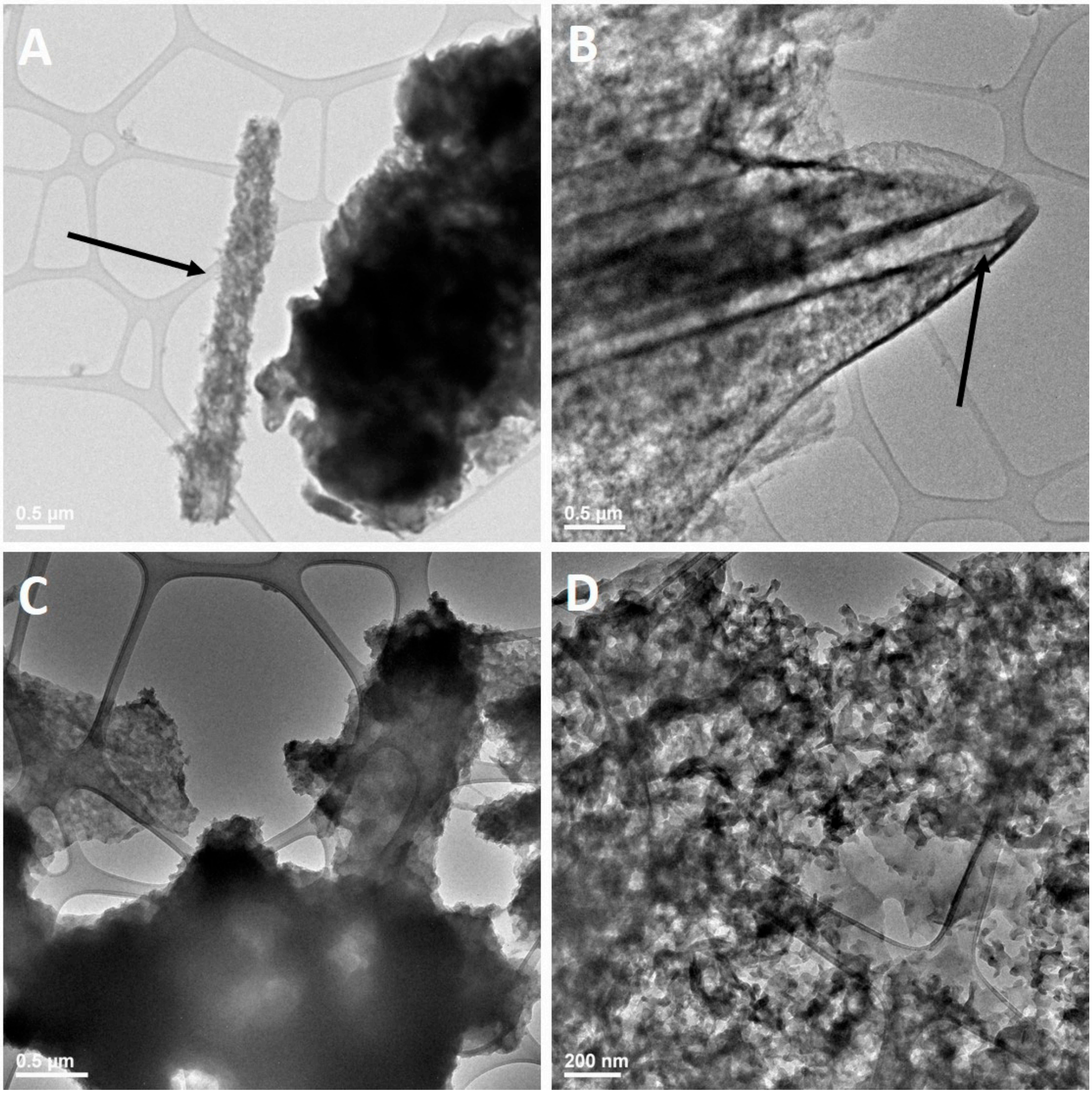

Figure S1 Transmission electron microscopy of g-CN (A-B) and g-CN-HT (C-D)

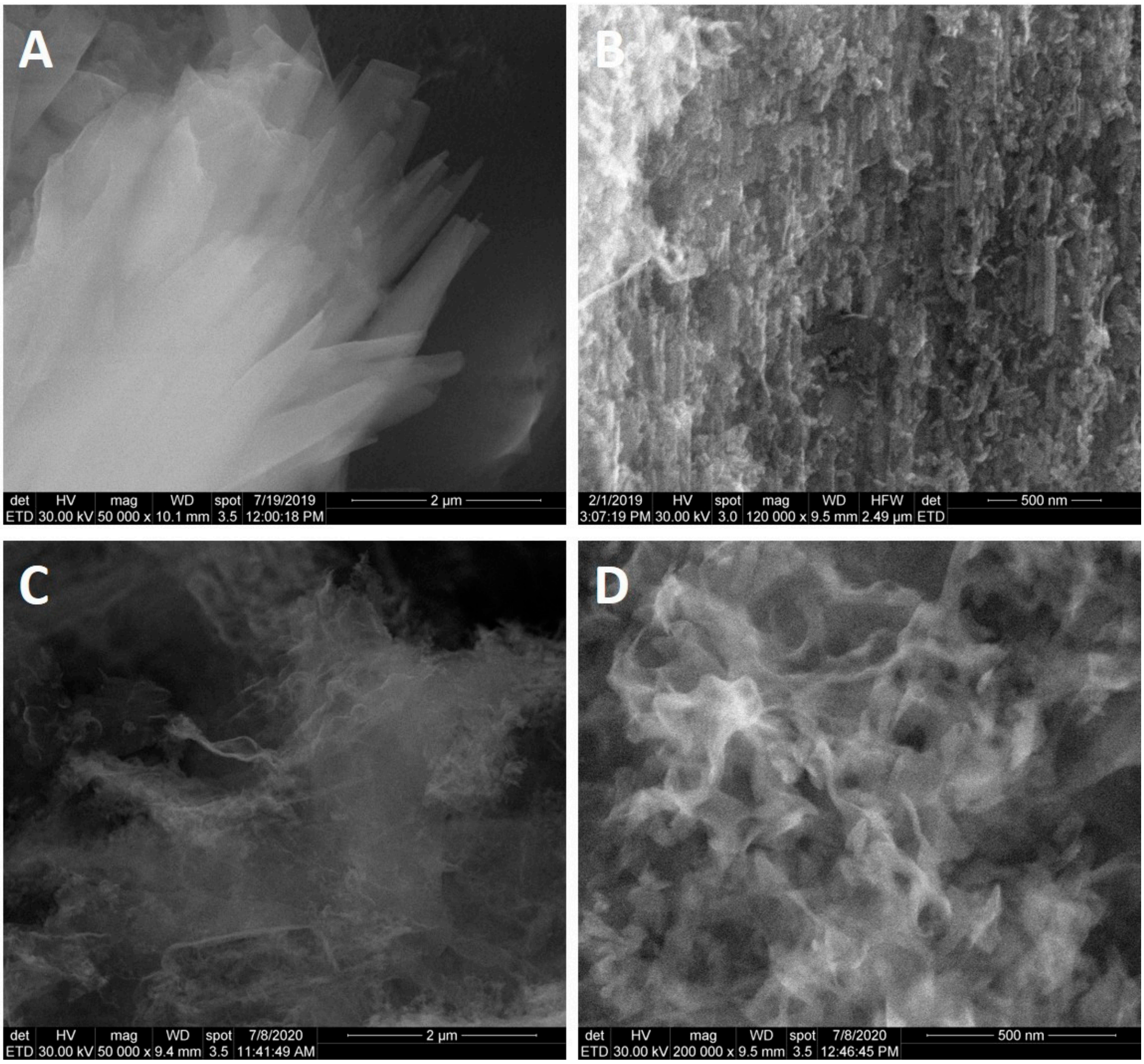

Figure S2 Scanning electron microscopy of g-CN (A-B) and g-CN-HT (C-D)

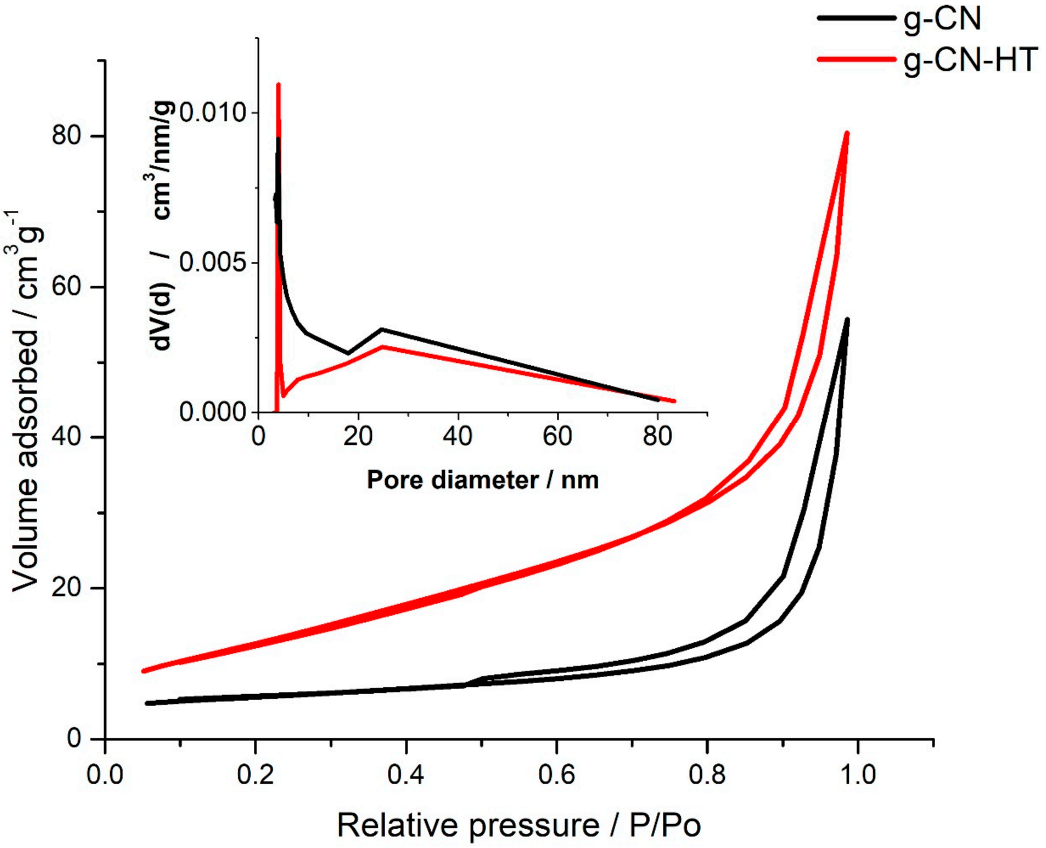

Figure S3 N_2 adsorption desorption isotherms for samples g-CN(black) and g-CN-HT(red)

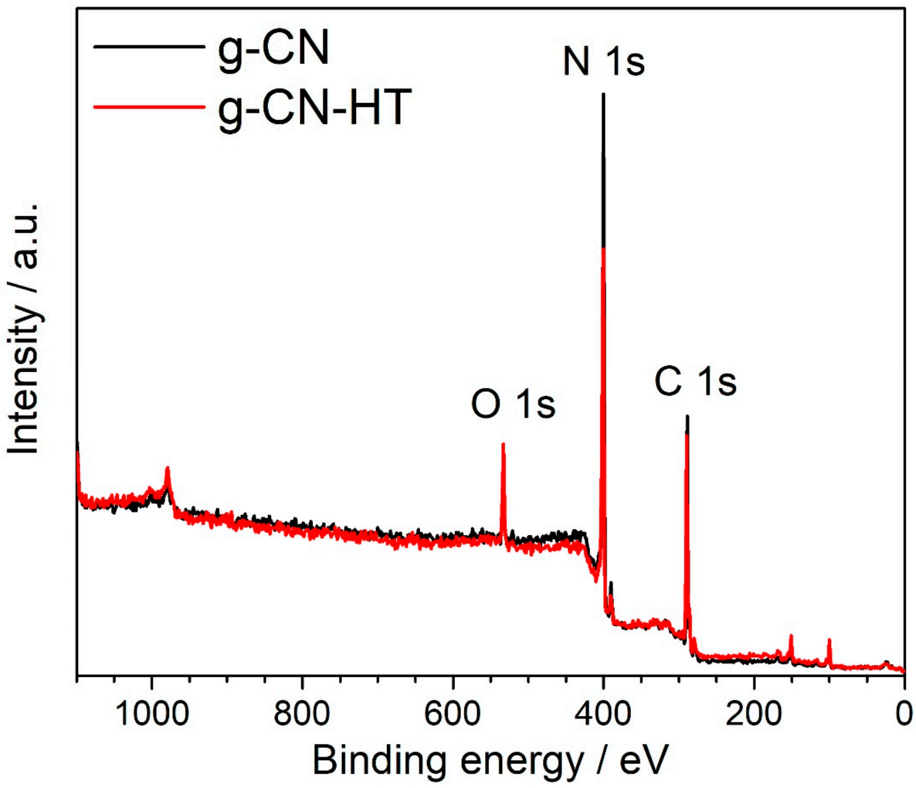

Figure S4 Wide spectrum X-ray photoelectron spectroscopy

Mott-Schottky equation:

$$\frac{1}{C^2} = \frac{2}{e\varepsilon\varepsilon_0 N_d}\left[\left(V - V_{fb}\right) - \frac{k_B T}{e}\right]$$

, where C is the specific capacitance (F cm^{-2}), e is the electron charge (=1.602 x 10^{-19} C), ε is the dielectric constant (i.e. 9.58 for carbon nitride[37]), ε_0 is the vacuum permittivity (=8.854 x 10^{-14} F cm^{-1}), N_d is the carrier density, V is the applied potential, V_{fb} is the flat band potential and k_BT is the well-known temperature dependent Boltzmann factor. The value of k_BT/e at room temperature is approx. 0.025 V which is considerably smaller than -1.78 and -1.69 V for g-CN and g-CN-HT, respectively.

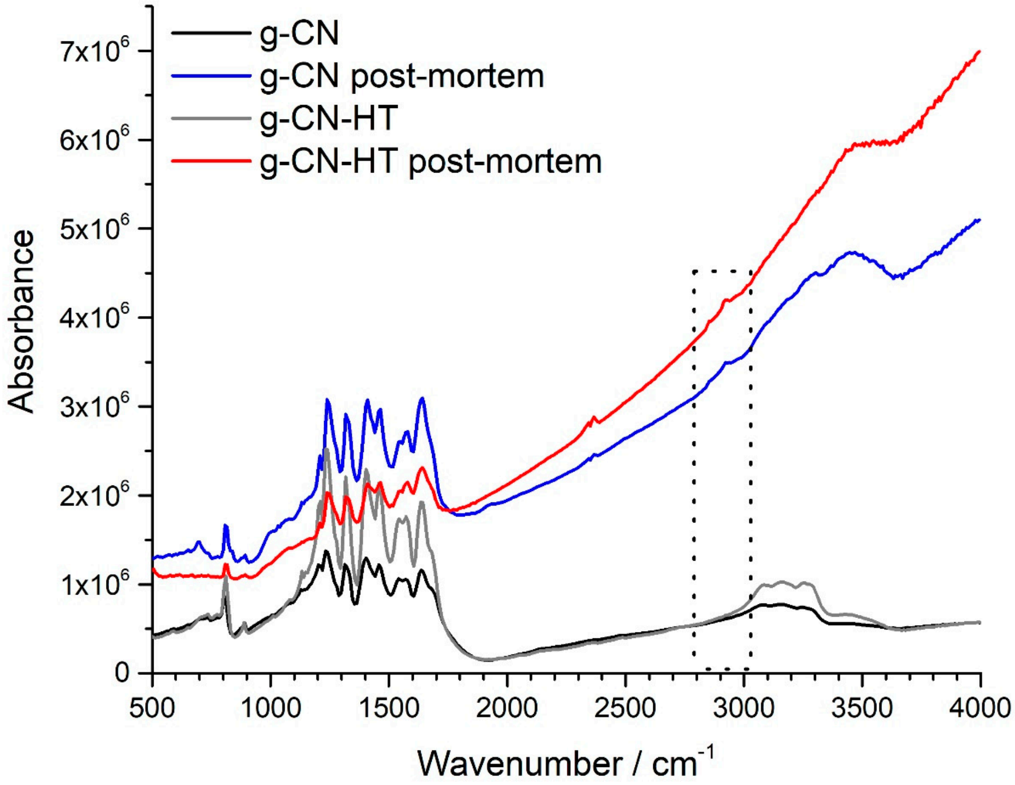

Figure S5 Raw FT-IR data

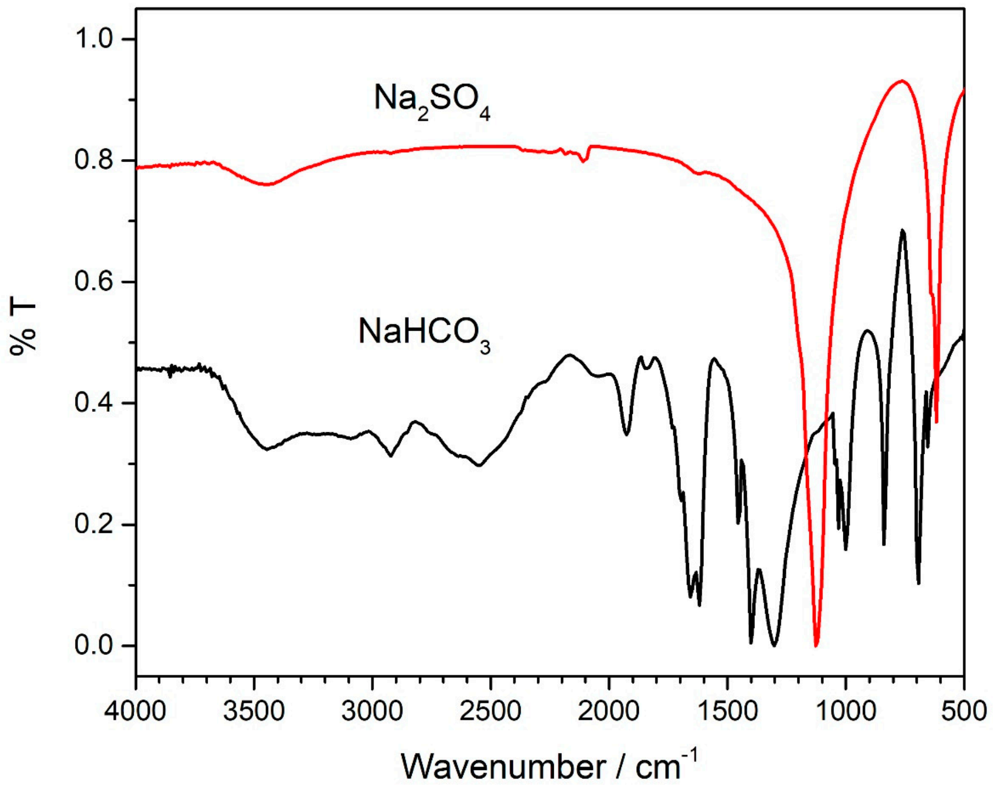

Figure S6 FT-IR relative transmittance of Na_2SO_4 and $NaHCO_3$

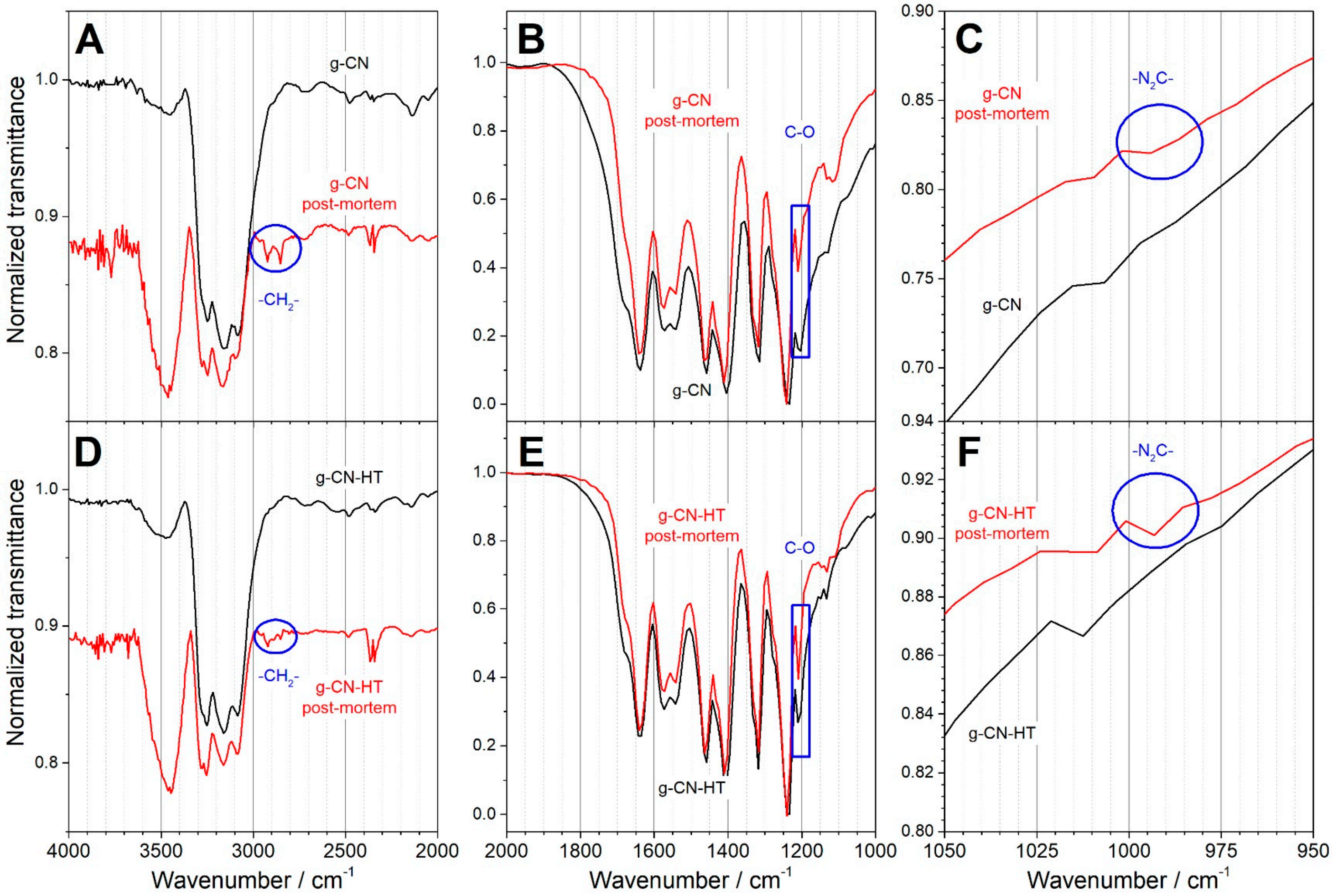

Figure S7 FT-IR relative transmittance of initial (black curves) and post-mortem (red curves)of g-CN and g-CN-HT. Blue circles shows the CH_2 specific vibration at 2853/2922 cm^{-1}. The red curves in A, C, D and F are y-offset by -0.1 on the y-axis .
